# Effect of Low Light Intensity With Supplemental Far‐Red Light on Growth, Yield and Quality of Broccoli Microgreens

**DOI:** 10.1002/fsn3.70542

**Published:** 2025-06-30

**Authors:** Shirin Shahkoomahally, Irma Ortiz, Xudong Zhu, Ellen R. Turner, Yanfang Li, Jianghao Sun, Tianbao Yang

**Affiliations:** ^1^ Food Quality Laboratory USDA‐ARS Beltsville Maryland USA; ^2^ Methods and Application of Food Composition Laboratory USDA‐ARS Beltsville Maryland USA

**Keywords:** ascorbic acid, *Brassica oleracea* var. *italica*, controlled environment agriculture, phytochemical, postharvest quality

## Abstract

One of the main obstacles to indoor agriculture is the high expense of lighting energy. The purpose of this study was to investigate how to grow broccoli microgreens under low light with higher yield and better quality. Broccoli seedlings were exposed to different photosynthetic photon flux densities (PPFD) ranging from 50 to 150 μmol/m^2^ s, along with supplemental far‐red (FR) light (20% of total photon flux density (TPFD)) at 50 and 75 μmol/m^2^ s. Broccoli grown under 50, 75, and 100 μmol/m^2^ s exhibited the highest fresh weight. As light intensity decreased, the hypocotyl length of broccoli microgreens increased. High chlorophyll, carotenoid, and anthocyanin contents were observed at 100 and 150 μmol/m^2^ s, while ascorbic acid and total phenolics were higher at 50 and 75 μmol/m^2^ s. The addition of FR light resulted in increased plant height, fresh weight, and antioxidant concentration. However, there was a significant decrease in total phenolic content. These results indicate that broccoli microgreen can adapt to low light with high yield and quality. Addition of FR under low light can further increase microgreen yield and plant height. Furthermore, postharvest quality and shelf‐life of microgreens under 50 and 75 μmol/m^2^ s were better than those under 100 and 150 μmol/m^2^ s. This research provides the platform for further managing microgreen growth under low light to reduce energy consumption for controlled environment agriculture (CEA).

## Graphical abstract

Broccoli microgreens grown under low light intensity (100 μmol/m^2^ s) displayed increased biomass, ascorbic acid, and total phenolic compounds. Adding far‐red light under low light further increased hypocotyl height and microgreen yield. Manipulation of low light intensity and far‐red light are effective to increase microgreen yield and quality while reducing the energy cost.

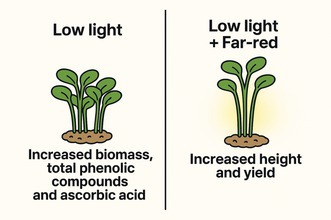

## 1 Introduction

Indoor farms, particularly those equipped with artificial light in plant factories, offer a stable, controllable, and secure environment for cultivating high‐value crops (Kozai 2018). Microgreens have become increasingly popular as a specialty vegetable in recent years, due to their short production cycle and high nutritional content (Aadil et al. 2013; Kozai 2018). Microgreens typically have higher concentrations of phytochemicals such as vitamin C, carotenoids, glucosinolate, phenolics, and flavonoids (Kozai 2018; Liu et al. 2021; Lu et al. 2021). Additionally, vitamin C, carotenoids, and flavonoids present in broccoli microgreens contribute to health benefits such as antioxidant, antibacterial, and anti‐inflammatory effects (Bian et al. 2018). It is noteworthy that the lighting conditions under which microgreens are cultivated can significantly impact the production and accumulation of these phytochemicals (Riga et al. 2019).

Light conditions, encompassing both quality and intensity, play a pivotal role in shaping the morphology and physiology of plants throughout their life cycle (Lefsrud et al. 2006; Lin et al. 2020). Light‐emitting diodes (LEDs) have emerged as a superior choice for optimizing crop production and quality in controlled environments, gradually replacing traditional lighting sources. Notably, white LED (W‐LED) light has gained popularity in indoor crop production (Zhang et al. 2020), particularly for leafy vegetables and microgreens, owing to its broad‐spectrum characteristics beneficial for plant growth (Meng and Runkle 2019; Nozue and Gomi 2018). The modification of light spectral quality is a crucial lighting approach for microgreens since it can influence morphology, yield, and nutrition, enabling great quality improvements (Rajan et al. 2019). There is little data available about how different LED light intensities affect the growth and nutrition of *Brassicaceae* microgreens, despite the fact that some studies (He et al. 2020; Kamal et al. 2020) have examined the impact of light quality on nutritional quality and bioactive compounds in microgreens.

Research indicates that varying light intensities influence the hypocotyl length and phytochemical content of microgreens. Gao et al. (2021) investigated the effects of various light intensities (30, 50, 70, and 90 μmol/m^2^·s) on the growth and phytochemical contents of broccoli microgreens. The broccoli microgreens under 50 μmol/m^2^·s had the largest fresh and dry weight. The contents of soluble protein, soluble sugar, free amino acid, flavonoid, and vitamin C were higher under irradiations of 70 μmol/m^2^·s. Therefore, 50 μmol/m^2^·s PPFD LED (red: green: blue = 1:1:1) might be the optimal light intensity for growth and 70 μmol/m^2^·s was good for the accumulation of phytochemicals in broccoli microgreens production. The light intensity between 50 and 70 μmol/m^2^·s might take account of growth and phytochemicals concentration (Gao et al. 2021). In another study, red pak choi and kohlrabi microgreens exhibited longer hypocotyl lengths under 110 μmol/m^2^·s compared to those grown under 220–545 μmol/m^2^·s (Samuolienė et al. 2013). Furthermore, Samuolienė et al. (2013) found that the vitamin C content of red pak choi and tatsoi microgreens increased under lower light intensity (110 μmol/m^2^·s). Red pak choi and tatsoi microgreens' total carotenoid content varied according to light intensity, with the highest levels seen at 330–440 μmol/m^2^·s and lower levels at both lower (110–220 μmol/m^2^·s) and higher (545 μmol/m^2^·s) intensities (Brazaitytė et al. 2015). Red pak choi exhibited much greater variation in total carotenoid content at different light intensities than did tatsoi. Higher light intensities enhanced the ferric reducing antioxidant power (FRAP) of both red and leafy vegetable amaranth microgreens, while the highest light intensities also markedly boosted the anthocyanin content of red amaranth microgreens (Meas et al. 2020). Furthermore, Kwack et al. (2015) found that sprouts of different vegetable species showed significant changes in their morphological properties when exposed to light intensities, varying from 0 to 100 μmol/m^2^·s.

Far‐red light can operate as a signal for plant growth, development, and metabolism by mediating important enzymes involved in related processes or metabolic pathways (Bae et al. 2017; Chen et al. 2016; Zhang et al. 2019; Zhen and van Iersel 2017). Plant biomass in seedlings, mature plants, and different ornamental crops is increased in indoor environments when far‐red light (11%–44% total photon flux density, TPFD) is added to RB‐LED lighting (Meng and Runkle 2019; Park and Runkle 2017; Zou et al. 2019). However, little is known about how far‐red light added to white LEDs (W‐LEDs) affects the morphology and phytochemicals of microgreens. Our recent study showed that FR light supplementation under low light intensity significantly increased the total glucosinolate content Li et al. (2025). According to findings (Ying et al. 2020a, 2020b), microgreen stem elongation was enhanced by supplemental low‐level far‐red light (20–40 μmol/m^2^·s) added at night to RB‐LED lighting or high‐level far‐red light (32% TPFD) added to RB‐LED lighting. Most phytochemical levels in lettuce grown indoors were unaffected by the addition of far‐red light (17% TPFD) to W‐LEDs. Nevertheless, the phytochemical concentration decreased when far‐red light and UVA were combined, indicating that far‐red light and UVA interaction affects accumulation of phytochemicals (He et al. 2021).

The typical light intensities ranging from 100 to 300 PPFD are commonly employed for microgreen production as indicated in the literature. However, there is a scarcity of studies focusing on the minimum PPFD required for microgreens (Zhang et al. 2020). Investigating lower light intensities is important because it can reduce electricity expenses related to microgreen production in CEA industry. By reducing the amount of light that microgreens receive, the dimming feature of LED lighting can help reduce power consumption and increase profitability by saving money on electrical energy. The effects of low light intensity on the development and phytochemical concentration of broccoli microgreens are not well understood. Furthermore, there are unknowns concerning the effects of adding far‐red (FR) light to white LEDs for the duration of indoor microgreen production. This study's main goal was to find out how light intensity—more precisely, less than 150 μmol/m^2^·s PPFD—affects the growth and accumulation of phytochemicals in broccoli microgreens, in the presence and absence of FR light.

## 2 Materials and Methods

### 2.1 Plant Material and Growth Conditions

Broccoli seeds ( *Brassica oleracea var. italica* cv. Ramoso Santana) were purchased from True Leaf Market Seed Company (Salt Lake City, UT, USA). The experiment was performed as previously described (Ortiz et al. 2024). The experiment was carried out in a controlled growth chamber (GC‐20 Bigfoot series, Biochambers, Winnipeg, MB, Canada). Seeds were sown at a density of 66,000 seeds/m^2^ on BioStrate felt hydroponic growing mats (Grow‐Tech Inc., Brentwood, TN, USA) in plastic trays (25 × 52 × 6 cm). Seeds germinated in darkness at 25°C and 90% relative humidity (RH) for 4 days to facilitates faster and more uniform germination (Finch‐Savage 2013). Deionized water (pH 5.8) was used to keep the mats moist throughout the experiment. On the fifth day after sowing, the seedlings were exposed to W‐LED lighting under a 16:8 h light: dark photoperiod regime and RH at 65%–75%. Fertilizer (FloraGro 2–1‐6, General Hydroponics Inc., Sebastopol, CA, USA) was delivered by foliar spray on day seven. Daily rotation of tray positions maintained an equal distribution of light and humidity over the chamber growth surface.

### 2.2 Light Treatments and Sample Collection

The adjustable LED panels (K5 Series XL750, Kind LED, Santa Rosa, CA, USA) with equalized white (peaking at 440 and 660 nm) and far red (730 ± 10 nm) were used as light sources. There were six light regimes: (1) W50 (50 μmol/m^2^·s PPFD); (2) W75 (75 μmol/m^2^·s PPFD); (3) W100 (100 μmol/m^2^·s PPFD); (4) W150 (150 μmol/m^2^·s PPFD); (5) W50 + FR (50 μmol/m^2^·s TPFD); (6) W75 + FR (75 μmol/m^2^·s PPFD). For FR supplement, 20% of TPFD was attributed to FR light. Light intensities of 50 and 75 μmol/m^2^·s PPFD or TPFD were regarded as low light in this study.

### 2.3 Growth Parameters Determination

Hypocotyl length of broccoli microgreens was determined by randomly selecting and measuring the stems of 10 plants per replicate. Subsequently, all broccoli microgreens for each replicate were cut 1 cm above the root and weighed using an electronic analytical balance (EAB) to obtain fresh weight (FW) and yield. Microgreens were then flash frozen in liquid nitrogen, placed in vacuum flasks, and sealed in a freeze‐dryer at −45°C until a consistent weight was achieved, to ascertain the dry matter weight (DMW). The dry matter percent was obtained by dividing DMW by FW and multiplying by 100.

### 2.4 Determination of Total Chlorophyll (TC) and Carotenoid (CC)

TC and CC measurements were performed following the method published previously (Ortiz et al. 2024). Briefly, fresh samples of broccoli microgreen cotyledons (0.1 g) were soaked in 40 mL of acetone 80% and left to incubate for 24 h at 25°C in the dark. The absorbance of the extract was calculated using a Bio‐Rad SmartSpec plus UV–Vis spectrophotometer (Hercules, CA, USA) at 663 nm (A663), 645 nm (A645) and 440 nm (A440).

$$\begin{array}{c} \text{Chlorophyll}\;a\;\text{content}\;\left(\mathrm{mg}/g\;\mathrm{FW}\right)\\ =\left(12.70\times A663-2.69\times A645\right)\times \text{dilution factor} \end{array}$$

$$\begin{array}{c} \text{Chlorophyll}\;b\;\text{content}\left(\mathrm{mg}/g\;\mathrm{FW}\right)\\ =\left(22.90\times A645-4.68\times A663\right)\times \text{dilution factor} \end{array}$$

$$\begin{array}{c} \text{Total chlorophyll}\;\left(\text{Chlorophyll}\;a+\text{Chlorophyll}\;b\;\text{content}\;\left(\mathrm{mg}/g\;\mathrm{FW}\right)\right)\\ =\left(8.02\times A663+20.20\times A645\right)\times \text{dilution factor} \end{array}$$

$$\begin{array}{c} \text{Carotenoid content}\;\left(\mathrm{mg}/g\;\mathrm{FW}\right)\\ =\left(4.70\times A440-2.17\times A663-5.45\times A645\right)\times \text{dilution factor} \end{array}$$

### 2.5 Determination of Ascorbic Acid Content (AAC)

With minor adjustments, the ascorbic acid content was ascertained using the method of Terada et al. (1978). A mixture of 6% HPO_3_ and 2 N acetic acid was added to 100 mg of freeze‐dried material, which was then frozen at −30°C. The samples were defrosted at room temperature and then blended for 1 min at 14,000 rpm using an Omni GLH tissue homogenizer equipped with a 10‐mm probe. The samples were centrifuged at 15,000 rpm for 20 min at 4°C. For each sample and ascorbic acid standard (5, 10, 20, 30, 40, or 50 μg/mL), a one mL aliquot was transferred into a glass test tube. Next, 2% dichloroindophenol (DCIP) was added, vortexed for 20 s, and incubated for 1 h at room temperature. Following incubation, 2% thiourea and 2% dinitrophenyl hydrazine (DNPH) were added to each test tube. The standards were kept at ambient temperature (25°C) while the sample tubes were vortexed and heated to 60°C for 3 h. Subsequently, the samples were cooled on ice for 3 h, and 2.5 mL of ice‐cold 90% sulfuric acid was added to each tube. The standard blank was mixed with 0.5 mL of 2% DNPH after each tube was vortexed. After transferring 250 μL of samples and standards into microplate wells, the absorbance was measured at 540 nm using a multi‐mode detection microplate reader (BioTek, SynergyTM2, USA). The standard curve was produced, and the ascorbic acid content was calculated for each sample using the linear regression equation.

### 2.6 Determination of Total Antioxidant Activity (TAA)

The ferric reducing antioxidant power (FRAP) assay, which was based on Benzie and Strain (1996), was used to calculate the ferric reduction activity using a method as in the previous publication (Ortiz et al. 2024). Briefly, freeze‐dried broccoli microgreens (200 mg) were added with 4 mL of 1% formic acid (v/v) in 80% MeOH. After extraction, for each sample and Trolox standard solution concentration, a 100 μL aliquot was combined with 900 μL of FRAP reagent. Next, using a multi‐mode detection microplate reader (BioTek, SynergyTM2, USA), the samples and standards were scanned at 595 nm.

### 2.7 Determination of Total Phenolic Compound (TPC)

The modified colorimetric Folin–Ciocalteu technique (Singleton and Rossi 1965) was used to calculate the total phenolic content as described in the previous published paper (Ortiz et al. 2024). Briefly, freeze‐dried material was added to 5 mL of extraction buffer, which contained water, methanol, and formic acid. The samples were incubated overnight at 4°C. The samples were centrifuged at 20,000 g for 20 min at 4°C the following day. Following centrifugation, 300 μL of the supernatant was combined with 300 μL of sodium carbonate (7.5%) and 300 μL of Folin–Ciocalteu reagent. After allowing the mixture to incubate at room temperature for 60 min in the dark, the absorbance at 760 nm was measured using a multi‐mode detection microplate reader (BioTek, SynergyTM2, USA).

### 2.8 Sensory Evaluation

With some adjustments, the sensory assessment of broccoli microgreens was conducted using the techniques outlined by Berba and Uchanski (2012) and Xiao et al. (2015). On harvest day, food‐grade linear low‐density polyethylene (LLDPE) bags were used to package three replicates with 10 g samples from each treatment group. The LLDPE bags weighed 8.0 g each and measured 16 by 12 cm with a thickness of 51 μm. The bagged samples were then stored at 40% relative humidity and 4°C, in the dark for 28 days. On 0, 4, 12, 20, and 28 days of storage, the freshness, color, off‐odor, and general quality of the microgreens were assessed using a 5‐point Descriptive Rating Scale as described by Ortiz et al. (2024).

### 2.9 Statistical Analysis

The data were evaluated for analysis of variance (ANOVA) and reported as mean ± standard error (*n* = 3 repetitions) using SPSS 26.0 software (Chicago, IL, USA). Tukey's test was used to compare the means, and differences were deemed significant at *p* < 0.05. Figures were drawn in R and Excel. R was used to create heat maps. The entire data set was examined using the R software's heatmap function. Minitab was used to determine the Euclidean distance and the full clustering algorithm among the samples.

## 3 Results

### 3.1 Effects of Light Intensity on Growth of Broccoli Microgreens

**FIGURE 1:**
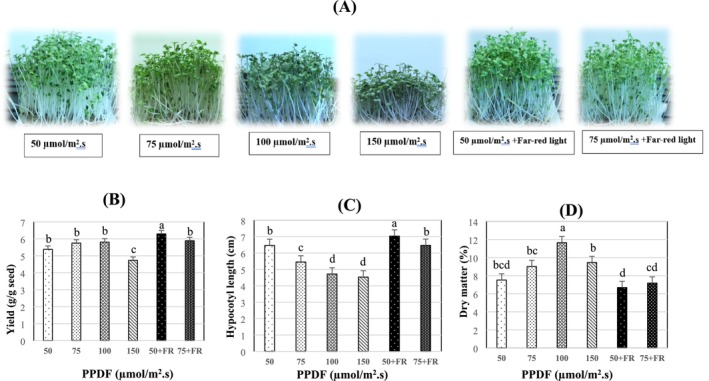
Light intensities affect broccoli microgreen growth and yield. (A) Plant images on harvest day; (B–D) fresh weight, hypocotyl length, and dry matter of broccoli microgreens. Broccoli (cv. Ramoso Santana) grown under different light intensities as shown under each chart were harvested on day 13. The lowercase letters in all figures represent the significant difference (*p* < 0.05). Error bars represent the standard error of the mean. Photosynthetic photon flux densities (PPFD), Far‐red light (FR).

Light intensity markedly impacted the growth of broccoli. As shown in Figure 1A, seedlings grown under 150 μmol/m^2^·s were shortest on harvest day, but decreased light intensity resulted in taller seedlings. A significant decrease in hypocotyl length (HL) was seen with an increase in light intensity (from 50 to 150 μmol/m^2^·s). When comparing those grown under 50 μmol/m^2^·s to those grown under 150 μmol/m^2^·s, HL was reduced by more than 30% (Figure 1C).

No significant difference in fresh weight was observed for microgreens grown under 50, 75 and 100 μmol/m^2^·s. However, the yield for all three of these treatments was noticeably higher than at 150 μmol/m^2^·s (Figure 1B). Dry matter percent of microgreens grown under 100 μmol/m^2^ was significantly higher than for other treatments (Figure 1D).

Supplementation of low light with 20% FR light increased canopy height by 21%. (Figure 1C). Fresh weight of plants receiving W50 + FR treatment was increased as compared to those receiving W50 treatment. However, there was no discernible difference in fresh weight between plants receiving W75 + FR and W75 treatments. Under WFR, broccoli microgreens produced a noticeably greater yield (6.5 g/g seed) and HL (7 cm) (Figure 1B,C). However, adding FR light decreased dry matter for broccoli microgreens by 27% compared with white light (Figure 1D).

### 3.2 Effects of Light Intensity on Total Chlorophyll (TC) and Carotenoid Content (CC) in Broccoli Microgreens

**FIGURE 2:**
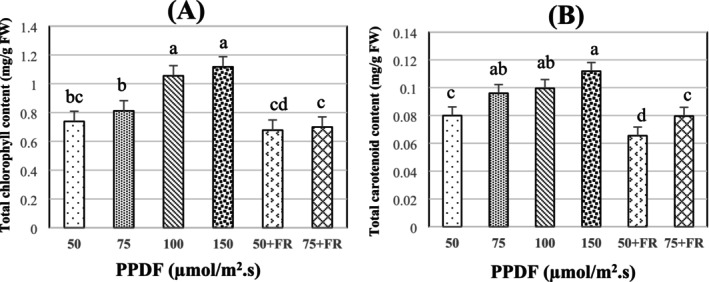
Total chlorophyll (A) and total carotenoid (B) content of broccoli microgreens (cv. Ramoso Santana) grown under PPDF levels of 50, 75, 100, 150, 50 + FR, and 75 + FR μmol/m^2^ s. The lowercase letters in all figures represent the significant difference (*p* < 0.05). Error bars represent the standard error of the mean. DW, dry weight; FR, far‐red light; FW, fresh weight; PPFD, photosynthetic photon flux densities.

There were variations in TC and CC across all light treatments. Both TC and CC increased by 57%, as the light intensity increased from 50 to 150 μmol/m^2^·s (Figure 2). Higher light intensity was therefore advantageous for increased TC and CC. Broccoli microgreens' total chlorophyll content did not substantially increase with increasing light intensity below 100 μmol/m^2^·s (Figure 2A). The amount of pigment accumulated was significantly impacted by the quality of the light. The TC of broccoli microgreens under WFR (50 + FR and 75 + FR) decreased by 8% and 17%, respectively, in comparison to W50 and W75. Furthermore, CC differences between W and WFR were considerable. Adding FR light to low light treatments decreased CC for broccoli microgreen by 33% (Figure 2B).

### 3.3 Effects of Light Intensity on Ascorbic Acid Content (AAC), Total Antioxidant Activity (TAA), Total Phenolic Compounds (TPC) of Broccoli Microgreens

**FIGURE 3:**
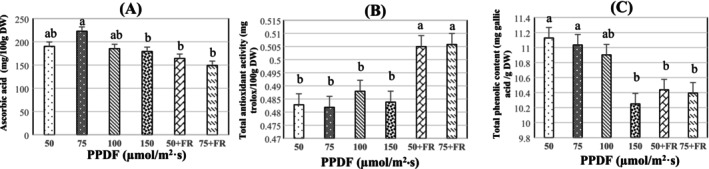
Ascorbic acid (A), total antioxidant activity (B) and total phenolic content (C) of broccoli microgreen (CV. Ramoso Santana) grown under PPFD levels of 50, 75, 100, 150, 50 + FR, and 75 + FR μmol/m^2^ s. The lowercase letters in all figures represent the significant difference (*p* < 0.05). Error bars represent the standard error of the mean. DW, dry weight; FR, far‐red light; FW, fresh weight; PPFD, photosynthetic photon flux densities.

AAC rose from 50 to 75 μmol/m^2^·s (Figure 3A), but then declined from 75 to 150 μmol/m^2^·s. At 75 and 150 μmol/m^2^·s, respectively, the highest (222.7 mg/100 g) and lowest (179.2 mg/100 g) concentrations were noted. The buildup of AAC was significantly impacted by the quality of the light. Adding FR light to the low light treatments decreased AAC for broccoli microgreens (Figure 3A). In broccoli microgreens grown under WFR (50 + FR and 75 + FR), the AAC dropped by 31% in comparison to white light (50 and 75 μmol/m^2^·s). Meanwhile, the quality of light did significantly influence TAA. The addition of FR light to low light intensity treatments resulted in a significant increase in TAA of 6% for broccoli microgreens (Figure 3B).

Phenolic content showed a decreasing trend with increasing light intensity treatment. The greatest phenolic decrease occurred with the increase of light intensity from 100 to 150 μmol/m^2^·s. Overall, the TPC in broccoli microgreens decreased by 10%, as intensity rose from 50 to 150 μmol/m^2^·s (Figure 3C). Higher light intensity was therefore not advantageous for the TPC accumulation in broccoli microgreens. The TPC was significantly impacted by the quality of the light. Supplementation of low white light with FR light significantly decreased TPC by 6% for broccoli microgreens (Figure 3C).

### 3.4 Effects of Light Intensity on Sensory Evaluation of Broccoli Microgreens

**FIGURE 4:**
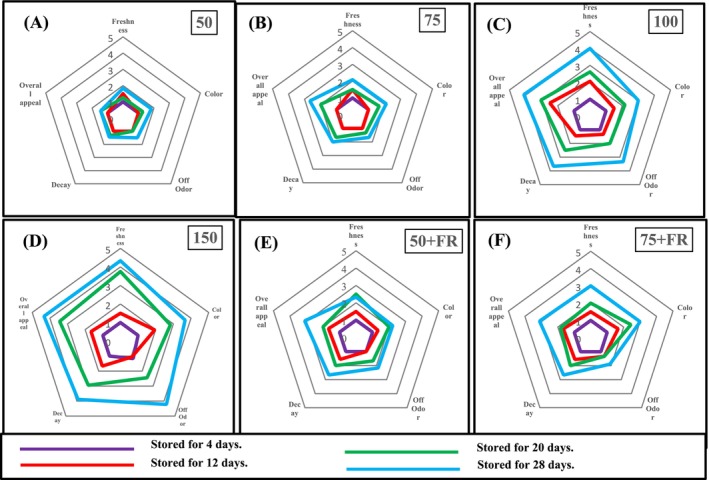
Spider diagrams of the sensory evaluation of broccoli microgreens (CV. Ramoso Santana) grown under PPFD levels of 50, 75, 100, 150, 50 + FR, and 75 + FR μmol/m^2^ s and stored for 4, 12, 20, and 28 days at 4°C. FR, far‐red light; PPFD, photosynthetic photon flux densities.

Spider diagrams (Figure 4) display the descriptive rating scale values for the five sensory qualities evaluated for broccoli microgreen samples subjected to the six different light treatments during 28 days of storage at 4°C. Sensory scores ranged from 1 to 4 on a scale of 1–5, with one being the highest quality and five the lowest. The sensory characteristics of the samples after 4 and 12 days of storage at 4°C maintained high quality with scores between 1 and 2 for all light intensities (Figure 4). No yellow leaves were seen on day 4 of storage. Yellow leaves were more common in microgreens produced under PPFDs of 150 and 100 μmol/m^2^·s after 12 days of storage than in those cultivated under 50 and 75 μmol/m^2^·s. By day 28, 25% of the samples grown under low light exhibited yellowing compared to 50% and 75% of samples grown under 100 and 150 W light treatments, respectively. Broccoli microgreens cultivated under 150 and 100 W light treatments only maintained excellent sensory quality for a 4‐day period, and by day 12, they had begun to steadily decline and were no longer salable by day 28 (Figure 4). Panelists reported worse overall quality (“poor”) for samples grown under 150 and 100 W on day 28 of storage than for those grown under 50 W (“very good”) and 75 W (“good”) (Figure 4).

All sensory characteristics were significantly impacted by the light quality. The addition of FR light reduced sensory quality of broccoli microgreens grown under 50 and 75 μmol/m^2^·s, which retained only “good‐fair” overall quality after 28 days storage. Broccoli microgreens grown under WFR (50 + FR and 75 + FR) were noted to have a brighter hue than under W (50 and 75 μmol/m^2^·s).

### 3.5 Heatmap Analysis

**FIGURE 5:**
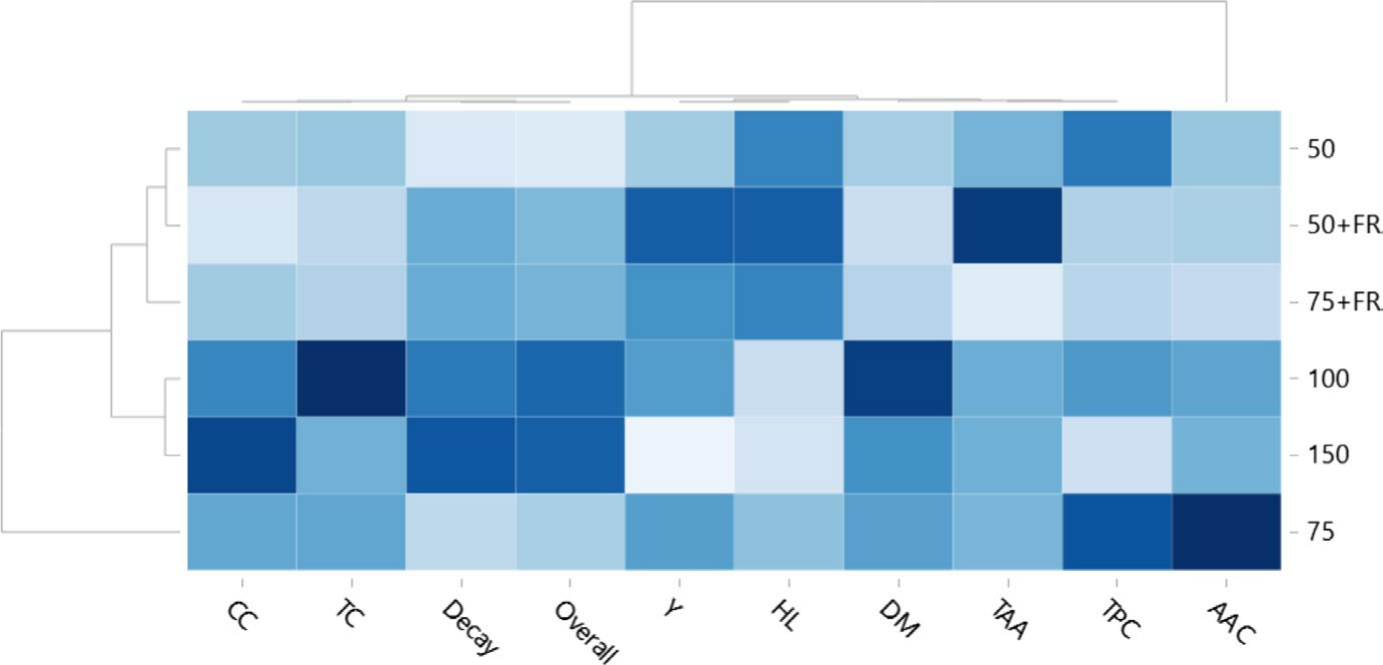
Heat map and hierarchical cluster analysis of the overall quality parameters of broccoli microgreens subjected to different light treatments. HL (hypocotyl length), Y (yield), TC (total chlorophyll content), decay, overall quality, CC (carotenoid content), DM (dry weight), TPC (total phenolic compounds), TAA (total antioxidant activity), and AAC (ascorbic acid content), FR (Far‐red light). The shades of light blue to dark blue indicate the relative value of the parameter.

A heatmap that integrated the response of the evaluated parameters provided a thorough understanding of how light intensity affected the growth and phytochemical content of broccoli microgreens (Figure 5). Regarding the reactions of the measured parameters, the clusters with 50 and 75 and 100 and 150 μmol/m^2^ s are the most similar to one another. In most of the investigated parameters, the lowest light intensity (50 μmol/m^2^ s) and highest light intensity (150 μmol/m^2^ s) exhibited opposite reactions. Compared to the 100 and 150 μmol/m^2^ s clusters, the 50 and 75 μmol/m^2^ s clusters displayed higher levels of HL and TPC. Meanwhile, the majority of the phytochemical parameters, including TAA and AAC, responded similarly in the 100 and 150 μmol/m^2^ s clusters. In broccoli microgreens, clusters 100 and 150 μmol/m^2^ s showed greater TC, CC, and DM. Additionally, phytochemicals were induced by cluster 100 μmol/m^2^ s more so than by the other three clusters. The HL and most phytochemical measures, including CC, TC, DM, TPC, and AAC, were greater under the 75 μmol/m^2^ s irradiation compared to those under 100 μmol/m^2^ s (Figure 5). The separation of white plus far‐red light (W + FR) from white light (W) was facilitated by the larger HL and lower phytochemical concentrations in the former compared to the latter. Lower incidence of decay and greater overall quality retention were the defining characteristics of lower light intensity treatments (50 and 75 μmol/m^2^ s), while higher light intensity treatments (100 and 150 μmol/m^2^ s) resulted in more rapid quality deterioration.

## 4 Discussion

### 4.1 Low Light Intensity and Supplement of FR on the Growth of Broccoli Microgreens

According to Zhang et al. (2020), broccoli microgreens are capable of thriving under artificial lighting with relatively low photosynthetic photon flux densities (PPFD), as low as 100 μmol m^−2^ s^−1^. In this study, treatments with even lower light intensities—specifically 50 and 70 μmol m^−2^ s^−1^—promoted vegetative growth, resulting in increased hypocotyl elongation, greater fresh yield, and lower dry matter content when compared to microgreens grown under 150 μmol m^−2^ s^−1^ (Figure 1). This stimulation of elongation growth was accompanied by a significant decline in total chlorophyll (TC) content under intensities below 100 μmol m^−2^ s^−1^ (Figure 2A), likely reflecting an adaptive strategy to prioritize vertical growth in search of additional light (Gao et al. 2021; Johnson et al. 2020; Kutschera and Briggs 2013). This chlorophyll suppression aligns with well‐established plant responses under low light conditions, wherein elongation is prioritized over pigment accumulation to optimize light interception. The observed promotion of hypocotyl elongation across multiple studies suggests this is a conserved photomorphogenic response beneficial for early‐stage development in microgreens. Nevertheless, while moderate reductions in light intensity may favor morphological traits linked to marketable appearance, extremely low light (e.g., 50 μmol m^−2^ s^−1^) led to the lowest dry matter accumulation in the present study. This corroborates previous findings indicating that insufficient irradiance limits carbon assimilation and biomass production in both broccoli (Gao et al. 2021) and lettuce (Fu et al. 2017). Thus, while certain low‐light conditions may enhance specific growth characteristics, there is a threshold below which photosynthetic limitations outweigh potential morphological benefits.

Artificial lighting significantly influences the morphological development of microgreens, particularly the hypocotyl, which is a key edible component of these crops (Zhang et al. 2020). Among the various environmental factors, light intensity plays a critical role in regulating hypocotyl elongation. Previous studies have demonstrated a clear inverse relationship between light intensity and hypocotyl length (HL). For instance, (Kwack et al. 2015) observed that as light intensity increased from 12.5 to 100 μmol m^−2^ s^−1^, HL significantly decreased across a range of sprout species including alfalfa, broccoli, clover, kohlrabi, radish, and red radish. Similar trends have been reported in mustard, mizuna, and kohlrabi sprouts, where HL declined progressively with increasing light intensity (Gerovac et al. 2016). Consistent with these findings, our current study revealed a 30% reduction in HL in broccoli microgreens when light intensity was elevated from 50 to 150 μmol m^−2^ s^−1^ (Figure 1C). The inhibition of hypocotyl elongation under higher light levels is likely mediated by reductions in endogenous gibberellin concentrations, a key plant hormone involved in shoot elongation (Binenbaum et al. 2018). In *Brassica* seedlings, increased light intensity has been shown to suppress gibberellin biosynthesis, thereby limiting elongation growth (Potter et al. 1999). It is plausible that similar hormonal regulation occurred in the present study, contributing to the observed morphological changes under elevated irradiance.

Interestingly, exposure to lower light intensity (50 μmol m^−2^ s^−1^) not only promoted hypocotyl elongation but also enhanced fresh weight in broccoli microgreens, mirroring trends reported for Chinese kale and cabbage microgreens (Gao et al. 2021; Liu et al. 2022). Additionally, Gao et al. (2021) reported increased chlorophyll accumulation in broccoli microgreens under 90 μmol m^−2^ s^−1^ white light, suggesting a light intensity threshold below which chlorophyll biosynthesis is limited. A positive correlation between pigment content and biomass has also been documented in Chinese kale and cabbage microgreens (Liu et al. 2022), further supporting the role of light intensity in optimizing both growth and nutritional quality. Moreover, our study found that higher light intensities (> 100 μmol m^−2^ s^−1^) were associated with elevated carotenoid levels (Figure 2B), likely due to their photoprotective function. Carotenoids help mitigate photooxidative stress under high light conditions by protecting the photosynthetic apparatus from excess excitation energy Jones‐Baumgardt et al. (2019).

As demonstrated in this study (Figure 1), the addition of far‐red (FR) light significantly enhanced biomass accumulation in broccoli microgreens under specific experimental conditions. This finding is particularly noteworthy given that far‐red light has traditionally been associated with the induction of shade‐avoidance responses and, in some cases, reductions in overall plant biomass (Chen et al. 2016; Xie et al. 2017). Although FR light lies outside the visible spectrum, it plays a critical role in photosynthetic processes (Pettai et al. 2005). In the present investigation, supplemental FR light (WFR) markedly increased the fresh weight of broccoli microgreens, suggesting improved photosynthetic efficiency and growth performance.

Mechanistically, far‐red light contributes to enhanced net photosynthesis by boosting the quantum yield of photosystem II while simultaneously reducing non‐photochemical quenching, thus facilitating more efficient use of absorbed light energy (Zhen and van Iersel 2017; Zou et al. 2019). This improvement in light‐use efficiency likely translates into greater assimilate production and biomass accumulation. In addition, FR light may promote morphological adaptations, such as shoot elongation and leaf expansion, which enhance light interception and canopy development—further contributing to increased biomass (Franjlin and Quail 2010; Franklin 2008; Meng et al. 2019; Park and Runkle 2017, 2018). These responses are hallmarks of the shade‐avoidance syndrome, typically triggered by a low red to far‐red (R:FR) light ratio. While these morphological changes are often considered disadvantageous in mature crops, they may be beneficial for microgreen production where shoot elongation and leaf enlargement are desirable traits for improving harvestable yield and visual appeal.

In the present study, the addition of far‐red (FR) light to white LED lighting resulted in significantly taller broccoli microgreens. This finding aligns with previous studies demonstrating that supplementing red and blue (RB) LED lighting with FR light enhances plant biomass, particularly in young plants (Meng and Runkle 2019; Park and Runkle 2017; Zou et al. 2019). Similar positive responses have been observed in indoor microgreen systems, where elevated levels of FR light promoted shoot elongation and increased biomass accumulation (Hooks et al. 2022; Ying et al. 2020b). These morphological changes are consistent with shade‐avoidance responses triggered by reduced phytochrome activity under low red to far‐red light ratios (Franklin and Whitelam 2005). As plants perceive FR‐enriched environments as shaded conditions, they often respond by elongating stems and petioles to outcompete neighboring plants for light. This elongation, while sometimes viewed as undesirable in mature crops, may offer practical advantages for microgreen production—particularly by facilitating easier mechanical harvesting. Moreover, the expansion of leaf area likely improved light interception, thereby supporting the observed increase in biomass (Park and Runkle 2017). Additionally, FR light may have enhanced photosynthetic efficiency via the Emerson effect, a phenomenon in which the simultaneous absorption of red and far‐red light boosts photosystem activity and overall carbon assimilation (Zhen and van Iersel 2017). Together, these physiological and morphological responses to FR light contribute to the improved growth and productivity observed in broccoli microgreens.

### 4.2 Phytochemical Changes in Broccoli Microgreens in Response to Low Light Intensity and FR

As a vital cofactor for numerous enzymes and an essential nutrient for human health, the accumulation of vitamin C in microgreens is a valuable response, particularly at lower light intensities, with peak concentrations observed around 75 μmol/m^2^·s. This phenomenon is of particular interest to Controlled Environment Agriculture (CEA) growers, as it suggests a beneficial trait for enhancing the nutritional profile of microgreens. For instance, microgreens of Chinese kale (Martín‐Cabrejas et al. 2008) and cabbage (Lin et al. 2020) exhibit higher vitamin C concentrations under 90 μmol/m^2^ s, likely due to the stress induced by these light conditions (Novickovas and Duchovskis 2019). This accumulation of antioxidants, including flavonoids, vitamin C, and carotenoids, plays a crucial role in mitigating the damaging effects of reactive oxygen species (ROS), which are often produced in plants under intense light exposure (Brazaitytė et al. 2015; Oh et al. 2009; Pérez‐López et al. 2018; Zhou et al. 2012). However, these findings is different from the results of this study as AAC and TPC were higher in lower light intensities than intense light intensity.

Broccoli microgreens treated with 50 + FR and 75 + FR displayed higher antioxidant activity compared to other light intensities, with the highest antioxidant levels observed at 50 + FR (Figure 3B). This increase in antioxidant content with higher light intensity does not align with trends observed in other *Brassica* microgreens (Samuolienė et al. 2013) and lettuce (Oh et al. 2009). Pérez‐López et al. (2018) suggested that it can be attributed to excess light energy at higher intensities, which exceeds the requirements for CO_2_ fixation in the Calvin cycle. This surplus energy can lead to the production of reactive oxygen species (ROS), which may damage photosystems (Hideg and Schreiber 2007). Antioxidants play a critical photoprotective role by interacting with the polar phospholipid heads at the water‐lipid interface of membranes, reducing the risk of oxidative damage caused by ROS (Erlejman et al. 2004).

Broccoli microgreens responded distinctly to White plus Far‐Red (WFR) light in this experiment. The application of WFR light resulted in a significantly higher yield (6.5 g/g seed) and greater hypocotyl length (7 cm) compared to other light treatments (Figure 1). However, WFR light also led to a notable reduction in the dry matter (DM) of broccoli microgreens, whereas White light increased DM (Figure 1). Additionally, significant differences in chlorophyll content (CC) were observed between plants grown under White (W) and WFR light. As light is a key environmental factor influencing leaf color, it is not surprising that FR light, known to reduce chlorophyll and carotenoid levels in lettuce, also affects leaf color in microgreens (Chen et al. 2016; Park and Runkle 2017; Sánchez‐Rangel et al. 2013).

This study investigated the effect of light spectra on the accumulation of health‐promoting phytochemicals in broccoli microgreens. Several metabolites, including total phenolic content (TPC) and ascorbic acid content (AAC), were high under low light (Figure 3). It should be noted that we observed the increase in total glucosinolate content (TGC) under 50 + FR light treatment in our recent study (Li et al. 2025). These results suggest the significant influence of adding FR under low light intensity on glucosinolate accumulation in broccoli microgreens. These metabolites are widely recognized for their health benefits, including protective effects against cancer and other chronic diseases (Keatinge et al. 2011; Wojdyło et al. 2007). A similar response in antioxidant levels was observed in thorn berries (*Rubus hongnoensis* Nakai) when FR light was added to white‐LED lighting (Oh et al. 2021). Therefore, adding FR under low light can boost broccoli microgreen fresh weight, total antioxidant activity, and total glucosinolate content.

## 5 Conclusions

This research shows that growing broccoli microgreens with optimal nutritional value requires the precise combination of light quantity and quality. Low light intensity can potentially decrease electrical energy costs of growing microgreens. With dimming options available for LED fixtures, power consumption can be lowered by decreasing the light intensity provided to microgreens. Lower electrical energy costs can increase profits. It has been discovered that broccoli microgreens benefit from light intensities lower than 100 μmol/m^2^ s for increased biomass and nutrients AAC and TPC, although carotenoid accumulation peaked in the range of 75–100 μmol/m^2^ s PPFD. In particular, the microgreens grown under 50 to 100 μmol/m^2^ s PPFD showed the highest yield, while the dry matter was observed to peak more narrowly at 100 μmol/m^2^ s. Between 50 and 100 μmol/m^2^ s of light, there may be a balance between boosting phytochemical concentrations and encouraging development. Future research should focus on investigating combined treatments of suitable light intensity and photoperiod to increase phytochemical contents without impeding the growth of microgreens. In addition, adding FR light to white LED light increased broccoli microgreen hypocotyl length and biomass. Quantities of specific phytochemicals, such as overall glucosinolate content, were increased by FR light (Li et al. 2025). Therefore, manipulation of low light intensity and adding FR can be an effective approach for growers to improve yield and nutritional quality of microgreens while reducing the energy cost.

## Author Contributions

**Shirin Shahkoomahally:** conceptualization (supporting), data curation (equal), formal analysis (equal), investigation (equal), methodology (equal), validation (equal), visualization (equal), writing – original draft (lead), writing – review and editing (equal). **Irma Ortiz:** conceptualization (supporting), data curation (equal), formal analysis (equal), investigation (equal), methodology (equal), validation (supporting), visualization (equal), writing – review and editing (supporting). **Xudong Zhu:** data curation (supporting), investigation (supporting), methodology (supporting), writing – review and editing (supporting). **Ellen R. Turner:** data curation (supporting), investigation (supporting), methodology (supporting), writing – review and editing (supporting). **Yanfang Li:** formal analysis (supporting), investigation (supporting), writing – review and editing (supporting). **Jianghao Sun:** formal analysis (supporting), investigation (supporting), supervision (supporting), writing – review and editing (supporting), writing – review and editing (supporting). **Tianbao Yang:** conceptualization (lead), formal analysis (supporting), funding acquisition (lead), investigation (supporting), methodology (supporting), supervision (lead), writing – review and editing (equal).

## Conflicts of Interest

The authors declare no conflicts of interest.

## Data Availability

The data that support the findings of this study are available from the corresponding author upon reasonable request.
